# (Don’t) Look at Me! How the Assumed Consensual or Non-Consensual Distribution Affects Perception and Evaluation of Sexting Images

**DOI:** 10.3390/jcm8050706

**Published:** 2019-05-17

**Authors:** Arne Dekker, Frederike Wenzlaff, Anne Daubmann, Hans O. Pinnschmidt, Peer Briken

**Affiliations:** 1 Institute for Sex Research and Forensic Psychiatry, University Medical Center Hamburg-Eppendorf, 20246 Hamburg, Germany; f.wenzlaff@gmail.com (F.W.); briken@uke.de (P.B.); 2 Institute of Medical Biometry and Epidemiology, University Medical Center Hamburg-Eppendorf, 20246 Hamburg, Germany; a.daubmann@uke.de (A.D.); h.pinnschmidt@uke.de (H.O.P)

**Keywords:** eye tracking, non-consensual image sharing, intimate images, objectification, objectifying gaze, rape myth acceptance, sexting

## Abstract

The non-consensual sharing of an intimate image is a serious breach of a person’s right to privacy and can lead to severe psychosocial consequences. However, little research has been conducted on the reasons for consuming intimate pictures that have been shared non-consensually. This study aims to investigate how the supposed consensual or non-consensual distribution of sexting images affects the perception and evaluation of these images. Participants were randomly assigned to one of two groups. The same intimate images were shown to all participants. However, one group assumed that the photos were shared voluntarily, whereas the other group were told that the photos were distributed non-consensually. While the participants completed several tasks such as rating the sexual attractiveness of the depicted person, their eye-movements were being tracked. The results from this study show that viewing behavior and the evaluation of sexting images are influenced by the supposed way of distribution. In line with objectification theory men who assumed that the pictures were distributed non-consensually spent more time looking at the body of the depicted person. This so-called ‘objectifying gaze’ was also more pronounced in participants with higher tendencies to accept myths about sexual aggression or general tendencies to objectify others. In conclusion, these results suggest that prevention campaigns promoting ‘sexting abstinence’ and thus attributing responsibility for non-consensual distribution of such images to the depicted persons are insufficient. Rather, it is necessary to emphasize the illegitimacy of the non-consensual distribution of sexting images, especially among male consumers of the material.

## 1. Introduction

Sexting, the sending of intimate or explicit personal pictures, videos, or texts [1], has become common practice within different age groups [2,3,4,5]. Definitions vary, and the confusion of consensual and non-consensual sexting proves to be a central conceptual problem. [6,7]. While consensual sexting refers to the purposeful, active, and often pleasurable sending of one’s own images, the non-consensual sharing of sexting images happens against the will or without the knowledge of the person depicted [8]. This non-consensual sharing is one of the most frequently discussed risks in the context of sexting [9,10,11,12,13,14,15,16,17,18]. If sexting images are forwarded against the will of the person depicted (e.g., in their circle of friends) or published on the internet, this poses a serious risk to mental health. Situations in which victims are exposed to public humiliation and online bullying can lead to grave psychosocial consequences, in some cases even suicide [3,7].

Not only in the public debate but also in ‘sexting abstinence’ campaigns [19], sexting, in general, is deemed dangerous [20]. Not differentiating between consensual and non-consensual sexting can lead to victim blaming if the depicted producers of the images are held responsible for the unintended dissemination [7]. This mechanism has been criticized in the theoretical context of ‘rape culture’ [21,22,23] and linked to the broader concepts of ‘sexual objectification’ [24,25,26,27] and ‘rape myth acceptance’ [26,28,29]. Objectification theory postulates that in western societies women are sexually objectified, treated as objects and are only considered worthy to the extent that their bodies give pleasure to others [29] (for reviews [28,30]). Sexual objectification can be seen as a continuum ranging from acts of violence to subtler acts such as objectifying gazes [30,31]. These gazes, conceptualized as visually inspecting (sexual) body parts, have been empirically demonstrated using eye-tracking technology [32]. Additionally, people who sexually objectify others have been shown to be more likely to accept rape myths [24,25], which serve to normalize sexual violence, e.g., through victim blaming (for reviews [27,33]). These subtle myths have been conceptualized as cognitive schemes [34] and demonstrated to influence eye movements [35,36].

Although research has evolved around non-consensual sexting and its correlates [7,9,20], little effort has been conducted to investigate reasons for consuming such images. The question arises why people consume non-consensual sexting material when mere comparisons with consensual material do not reveal apparent differences in image content. Is there a specific attraction in the non-consensuality itself, at least for some of the consumers? Against this background, we experimentally investigate the question of how the supposed way of distribution (consensual vs. non-consensual) influences the perception of sexting images. Thus, the study promises important findings for future prevention efforts.

In accordance with the objectification theory we expect differences in evaluation and perception of sexting images depending on their supposedly consensual or non-consensual forwarding. In line with previous research, we argue that increased objectification is associated with higher attractiveness ratings of the objectified person [37] and a more pronounced objectifying gaze [32]. We further hypothesize that supposedly non-consensually forwarded images are considered as more intimate and their further distribution as more unpleasant. Overall tendencies for other objectification and higher rape-myth acceptance are also expected to increase objectification.

A large part of the scientific literature on sexting focuses on the behavior of adolescents. This may reflect widespread societal fears, but, in fact, sexting experience is significantly higher among adults than among adolescents. In a current systematic review [3] the prevalence estimate of studies of adolescents sending messages containing sexually suggestive texts or photos was found to be 10.2% (95% CI (1.77–18.63)), while the estimated mean prevalence of studies of adults was 53.31% (95% CI (49.57–57.07)). Against this background, and also because the present experimental study does not focus on a representative image of the user population, we have decided to examine a sample of adults. We assume that the mechanisms shown are comparable in adolescents, but this must be demonstrated by future research.

## 2. Materials and Methods

### 2.1. Participants

A total of 76 participants (57% female, M_age_ = 31.99, SD_age_ = 10.28) were recruited via university newsletters. They were informed about the tasks and the stimulus content but were kept naïve to the full purpose of the experiment. Participants provided written consent to study participation. No compensation was given. The ethics committee of the state chamber of psychotherapists of Hamburg (Psychotherapeutenkammer Hamburg) approved the study protocol of the present study (03/2015-PTK-HH).

### 2.2. Stimuli and Apparatus

Volunteers personally known to the authors but unknown to the study participants provided 14 semi-nude sexting images [38]. One additional image per gender was obtained from freely available internet sources for public presentation purposes, resulting in a set of 16 pictures (50% female).

Stimulus presentation and data collection were conducted on a 22-inch widescreen monitor (1680 × 1050 pixels) using SensoMotoric Instruments (SMI GmbH, Teltow, Germany) software ExperimentCenter^TM^. A remote eye tracker (SMI, RED system) recorded eye movements at 120 Hz from 50 cm viewing distance using a head-chin rest.

### 2.3. Questionnaires

Individuals’ objectification of others was assessed using a German translation of the modified version of the Self-Objectification Questionnaire [39] for other objectification (Other Objectification Scale, OOS [40]). The scale consists of 10 body attributes, five competence-based (i.e., strength) and five are appearance-based (i.e., physical attractiveness). Participants were asked to rank how important they perceive each attribute (10 = “most important”; 1 = “least important”) separately for men and women. Possible scores range from −25 to 25 with higher scores indicating higher levels of objectification.

Participants further completed an 11-item short version of the German Acceptance of Modern Myths About Sexual Aggression Scale (AMMSA) [41] which had been used successfully in other eye tracking studies already [35,36]. Each item was rated on a 7-point scale (1 = “completely disagree”; 7 = “completely agree”).

### 2.4. Procedure

Participants read an introductory text stating that the study aimed to understand more about the evaluation of sexting images. Depending on the condition, picture distribution was either described as voluntary (consensual condition) or as unwanted, against the will of the depicted person (non-consensual condition). The manipulation was strengthened by asking participants to state three feelings the image distribution could have evoked in the depicted persons. Following, participants saw the images three times with different tasks. Pictures were randomized within blocks, starting with the male images. Pictures were presented individually on full screen for 5 seconds, preceded by a black fixation cross on the left side shown for 1 second. The first task was to freely view the pictures. Second, participants rated the sexual attractiveness of the depicted person. For the third task, participants were asked to evaluate how intimate they considered the image content and how unpleasant further picture distribution would be for the depicted person (ranging from 1 = “not at all …”; 7 = “very …”). After completion of the sociodemographic information, and the questionnaires, participants were thanked and debriefed.

### 2.5. Data Reduction and Data Analysis

To account for repeated measures made on the same subject, a mixed model approach was employed. We examined the fixed effects of the independent variables condition (consensual vs. non-consensual distribution), gender (women vs. men), image gender (female vs. male images), of their three and two-way interactions and of the OOS score and AMMSA score on the ratings of (1) sexual attractiveness, (2) intimacy of image content, and (3) perceived unpleasantness of picture distribution. Random intercepts were assumed for participants. We report the marginal means and their 95%-confidence intervals. We report the results of the final models after a backward elimination of the non-significant effects according to Kleinbaum et al. [42]. All statistical tests were two-tailed (α = 0.05). 

The eye tracking data were analyzed using the same model as described above with the objectifying gaze as the dependent variable. The objectifying gaze was operationalized as the relative time spent looking at the body compared to the time spent looking at faces [32]. We created two areas of interest (AOI) on each image, one containing the head and the other containing all the rest of the body. The total dwell time for both AOIs, i.e., the overall time viewing the person depicted, was set to 100%. For the following analysis, we focus on the percentage of that time directed at the body. Accordingly, an increase in viewing time on the body always results in a decrease of dwell time on the face, since both values always add up to 100%. So a stronger objectifying gaze refers to relatively longer viewing time on the body and shorter viewing time on the face.

Computations were done using the GENLINMIXED (Generalized linear mixed model) routine of SPSS version 22 (IBM Corporation, Armonk, NY, USA) and eye tracking data reduction was realized using the standard settings of BeGaze^TM^ (SMI, Teltow, Germany), providing gaze information such as duration (dwell time).

## 3. Results

### 3.1. Participants

Prior to data analysis participants were excluded due to poor recordings (*n* = 5), non-heterosexual orientation (*n* = 3), or due to inadequate responses to the manipulation check (*n* = 10) as rated by four independent raters. A total of 58 participants (57% female, M_age_ = 31.45, SD_age_ = 10.18) remained for data analysis (see Table 1). Table 1 also shows the means of participants’ AMMSA and OOS scores. In this context, it is particularly important that the mean values of the two study groups do not differ.

### 3.2. Ratings

Separate models were conducted for each of the three explicit ratings, namely sexual attractiveness of the person depicted, perceived intimacy of the image content, and unpleasantness of further distribution. Only the significant effects of the final models are reported here.

For attractiveness ratings, we did not find that condition (consensual vs. non-consensual distribution; see Table 2) had any effect. We did, however, find that gender had an effect as well as an interaction effect between participant gender and image gender. Overall, men rated the images of men as more attractive (M = 4.17, SE = 0.32) than women did (M = 3.02, SE = 0.31; *t*(924) = 3.25, *p* < 0.001). Women also rated the images of men as less attractive than images of women (M = 4.46, SE = 0.32, *t*(924) = 9.36, *p* < 0.001). No other effects reached significance.

Concerning the intimacy ratings, we found an interaction effect between condition and gender (*p* = 0.008, see Table 2). Pairwise contrasts revealed that women who assumed non-consensual distribution regarded the images as more intimate (M = 4.86, SE = 0.25) than women who assumed consensual distribution (M = 4.56, SE = 0.26; *t*(924) = 2.58, *p* = 0.01).

Analyzing influences on how unpleasant further distribution was considered for the depicted person, we found that condition (consensual vs. non-consensual distribution; *p* < 0.001) had a highly significant effect (see Table 2). Pairwise contrasts revealed that participants assuming non-consensual sharing considered further distribution as more unpleasant (M = 4.63, SE = 0.28) than participants who assumed consensual sharing (M = 4.26, SE = 0.28; *t*(924) = 3.74, *p* <.001). We also found an interaction effect between gender and image gender. Women rated the unpleasantness of further distribution lower for images of men (M = 4.08, SE = 0.40) than male participants did (M = 4.41, SE = 0.40; *t*(924) = 2.50, *p* = 0.013). Furthermore, the AMMSA score reached significance (coefficient = −0.13, *p* = 0.002), indicating that the higher participants scored on the AMMSA-scale, the less unpleasant they considered picture distribution for the depicted person.

### 3.3. Eye Tracking Analysis

**Figure 1:**
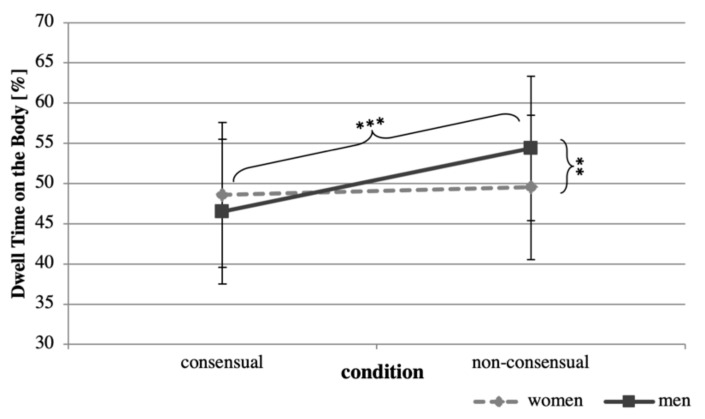
Estimates of the mean proportion (and standard error) of dwell time spent on the body by condition and gender. *** *p* < 0.001; ** *p* < 0.01.

Regarding eye movements, we were interested in the objectifying gaze, operationalized as the relative time viewing the body. We found a significant interaction of condition and gender (*F*(1,834) = 8.36, *p* < 0.001). Men in the non-consensual condition demonstrated a stronger objectifying gaze as they looked significantly longer at bodies *(*M = 54.37, SE = 8.99) than men in the consensual condition (M = 46.52, SE = 9.01; *t*(834) = 4.25, *p* < 0.001) (see Figure 1). Within the non-consensual condition, men also demonstrated the objectifying gaze more than women did, spending more time looking at bodies than women did (M = 49.53, SE = 8.97; *t*(834) = 3.07, *p* = 0.002). Notably, there was no such gender difference within the consensual condition (*p* > 0.05).

The effects of the OOS score and the AMMSA score were significant (*p* < 0.001), indicating that relative dwell time on the body increases for higher scores. In other words, this reveals a more pronounced objectifying gaze for higher tendencies to objectify and accept myths about sexual aggression (see Table 3).

## 4. Discussion

We demonstrate that not only explicit ratings but also the implicit viewing behavior are influenced by the assumed consensual or non-consensual distribution of sexting images. 

### 4.1. Image Evaluations

Participants who assumed the non-consensual distribution of a sexting image, namely the sharing against the will of the person depicted, rated the further distribution of the images as more unpleasant. This finding demonstrates that not only the picture content itself or personal feelings about sexting but also the surrounding information is considered when estimating the unpleasantness of further picture distribution. Interestingly, women rated the unpleasantness of distribution lower for images of men than male participants did. Seeing images of other men, the risk saliency of becoming a victim and having one’s images shared non-consensually might have increased for men, leading to higher ratings of unpleasantness. Due to the common stories of non-consensual sexting involving women, female participants might be aware of personal risks at any time independent of the condition. As the potential consequences of forwarding are more severe for women [43,44], female participants might consider further forwarding as less unpleasant because of the less severe consequences for men. However, it is important to note that the images of men and women should not be compared directly with each other in this study as picture compositions varied. Men were usually posing less sexually than women, which is due to the naturalistic creation of the images, but likely influences the ratings of unpleasantness.

Overall, higher general rape myth acceptance led to lower ratings of perceived unpleasantness of further distribution in both conditions. Higher endorsement of rape myths is indicative of a higher likelihood of victim blaming, which is in line with the common risk discourses on sexting [7,12,22,45]. Accordingly, considering non-consensual sharing a risk inherent in sexting allows minimizing the expected level of unpleasantness of further distribution. The depicted person is deemed responsible for having taken the image to begin with and hence either stupid or reckless. In other words, the estimated unpleasantness decreases when victim blaming increases. This is crucial as this pattern is not only typical for cases of revenge pornography [46] but also for other forms of sexual harassment [26,47] and has even found its way into ‘sexting abstinence’ campaigns [20]. Concerning the perceived intimacy of the images, women assuming non-consensual distribution rated the images as more intimate for both genders than women assuming consensual sharing. Men, however, did not differ between the consensual or non-consensual distribution of images of either men or women. This could be attributed to the fact that women are more likely to be victims of non-consensual sexting [3] and to be victimized in general in most forms of online gender-based violence [19,48]. Being aware of the potential personal risk might make women more sensitive to the intentions of the depicted person and violations of privacy.

Unlike expected, the assumed way of distribution did not affect how participants rated the sexual attractiveness. Previous research linking objectification and attractiveness ratings presented women in casual wear and the same women in bikinis [46]. Such a strong manipulation allows for large differences between conditions. Using the same semi-nude images in both conditions as done in our study might not have been a strong enough manipulation to affect explicit attractiveness ratings. The exhibited interaction effect between gender and image gender, more precisely higher ratings of male images by men, is likely due to factors inherent in the images and not the context. Therefore, we do not consider them as relevant for this study.

### 4.2. The Objectifying Gaze

The objectifying gaze, defined as the relative amount of time looking at the body, was influenced by condition and participant gender. Men assuming non-consensual distribution displayed the objectifying gaze more than men assuming voluntary sharing and more than women assuming either manner of distribution. Hence, we were able to demonstrate for the first time that the supposed way of distribution influences how participants look at images and how strongly they display the objectifying gaze. Previous research suggests that especially women are sexually objectified in the media [26,49,50] and during interpersonal interactions [51,52]. The objectifying gaze has been linked to negative social perceptions, dehumanization, and self-objectification [53,54,55]. While an appearance-focus in women has been linked to negative social perceptions [54,55] and severe mental health problems [55], no comparable research on men exists.

Although mostly discussed for men, women are thought to have internalized the objectifying gaze so much that they demonstrate it toward other women as well [56]. However, in our study, only men assuming non-consensual distribution differed from the other participant groups, albeit unaffected by the gender of the depicted person. Unlike other studies [57,58,59,60], we did not find systematic influences of image gender on viewing behavior. We suggest that our manipulation might have evoked other task demands that resulted in viewing patterns different from free viewing conditions, possibly covering influences of image gender [61]. In line with previous research, higher general tendencies to objectify others, as well as higher acceptance of rape myths, were related to a more pronounced objectifying gaze [35]. Numerous gender-specific functions and consequences have been reported for rape myths acceptance (for a review see [62]). Still, due to cultural changes, rape myths and sexist beliefs have become increasingly subtle as taken into consideration and measured by the acceptance of modern myth about sexual aggression scale applied here [63]. This study is the first to consider the influences of both biases on eye movements and suggests that subtle attitudes indeed affect viewing behavior. These influences and their implications should be further investigated in the context of sexual aggression.

### 4.3. Limitations and Future Research

Our study was conducted in the laboratory with well educated, heterosexual participants viewing images of young, attractive adults who were semi-nude, unlike in most severe cases of non-consensual image sharing [64]. Accordingly, the generalizability of our results needs further investigation. Future research has to take intersectional influences (e.g., skin color or age) into account, as these factors are relevant in the context of objectification [50]. Concerning participants, intersectionality is also important, as cultural influences regarding eye-movements [65], sexual objectification [66], and sexual harassment [67,68] have been found. Other reasons for fixating more on bodies (e.g., social comparison) or avoiding faces (e.g., shame) should be explored as well.

As mentioned above, in this study we have focused on adult participants for two main reasons: First, the prevalence of sexting among adults is actually higher than among adolescents. Secondly, we were not interested in a representative image of the user population, but in an experimental comparison of two equivalent groups. Nevertheless, it is possible that the correlations shown do not exist among adolescent users. For this reason, a replication of the present study with adolescent participants would be desirable.

Although we demonstrated that the supposed manner of distribution affects the perception of sexting images, qualitative research asking consumers of non-consensual sexting for their motives seems like an important step to further identify the beliefs behind such behavior, (e.g., the enjoyment of power) [69]. Another aspect is the perceived agency of the depicted person that might be decreased by non-consensual forwarding, which in turn could facilitate objectification. This idea needs further investigation.

Since everyday sexual objectification is common [70], it is crucial to examine and develop theories regarding possible outcomes and further explore the similarities between sexual assault and non-consensual pornography, or technology-facilitated violence in general.

As rapid changes of the technological landscape routinely link new types of specific behavior (e.g., non-consensual sexting) to existing theory (e.g., on sexual objectification) they can inform the creation of prevention programs [46,71]. The well-researched theory of ‘sexual double standards’ suggests that women’s sexuality is often perceived as pure and damageable through active desire, holding women responsible for protecting themselves from aggressive male sexuality [72,73]. This leads to the paradoxical position for women of experiencing social and cultural pressure to be sexy while simultaneously risking negative social consequences when portraying themselves in such manner online [74,75]. Considering the sexual double standard allows us to understand nonconsensual sexting as reaffirming stereotypical gender roles that place women under the control of men [53,55]. As girls are more likely to engage in sexualized self-presentations on social network sites and more attention is paid to their physical appearance than that of boys [76], gendered aspects need to be considered [17,77]. While arguments have been made to consider sexting as an empowering (social) media production [78,79] and to frame sexy appearance as a feminist act to counter the negative effects of objectification [80], this positive reframing carries the potential negative effect of normalizing unwanted sexual attention, which may outweigh the possible benefits of individual self-preservation [71].

## 5. Conclusions

In conclusion, we demonstrated that viewing behavior and evaluation of sexting images are influenced by their supposed consensual or non-consensual distribution. In line with objectification theory, an ‘objectifying gaze’ was more pronounced in men who assumed non-consensual picture distribution, meaning they spent a relatively longer time looking at the body of a depicted person. This ‘objectifying gaze’ was also more pronounced for participants with higher tendencies to accept myths about sexual aggression or general tendencies to objectify others. The results suggest that prevention campaigns that focus on a general message of sexting abstinence and thus attribute responsibility for non-consensual distribution of such images to the persons depicted are insufficient. Rather, it is necessary to emphasize the illegitimacy of the non-consensual distribution of sexting images, especially among male consumers of the material. This can be done, for example, in the context of school educational events, but there is also at least one example of an appropriate public prevention campaign: http://notyourstoshare.scot/. Only with these or comparable measures can the serious psychological consequences of public humiliation and online bullying be prevented in the long term.

## Figures and Tables

**Table 1:** Participant characteristics and questionnaire data.

	Condition	
	Consensual ^a^	Non-Consensual ^b^
Female (%)	52%	61%
Age (M, SD)	32.20 (11.75)	31.42 (9.16)
Age (Range)	21–68	19–59
AMMSA score (M, SD)	2.96 (1.33)	2.44 (0.90)
OOS score (of Women; M, SD)	4.58 (10.86) ^c^	−0.44 (10.16)
OOS score (of Men; M, SD)	0.67 (8.42)	−0.94 (9.67)

The means do not significantly differ between conditions (*p* > 0.08). OOS score = Scores on the Other Objectification Scale (Strelan and Hargreaves, 2005) separately for the objectification of women and of men; the possible range is from −25 (low objectification) to 25 (high objectification). AMMSA score = Scores on the 11-item short version of the Acceptance of Modern Myths About Sexual Aggression scale (Gerger et al., 2007); the possible range is from 1 (low acceptance) to 7 (high acceptance). ^a^ *n* = 25. ^b^ *n* = 33. ^c^ *n* = 24.

**Table 2:** Final models of the influences on ratings of sexual attractiveness, intimacy, and presumed unpleasantness of further distribution.

Dependent Variable	Independent Variable	*F*	*p* Value	Coefficient ^a^	95% Confidence Interval	
					Lower Limit	Upper Limit
Sexual Attractiveness	Gender	50.82	<0.001	−1.15	−1.39	−0.91
	Image Gender	4.34	0.038	0.38	−0.50	1.26
	Gender × Image Gender	36.89	<0.001	1.06	0.72	1.40
Intimacy	Condition	0.610	0.435	0.16	−0.09	0.42
	Gender	0.025	0.874	0.22	−0.01	0.45
	Group × Gender	7.029	0.008	−0.46	−0.80	−0.12
Unpleasantness	Condition	14.02	<0.001	−0.37	−0.56	−0.18
	Gender	1.47	0.225	−0.34	−0.60	−0.07
	Image Gender	0.52	0.473	0.18	−0.93	1.28
	Gender × Image Gender	5.41	0.020	0.44	0.07	0.82
	AMMSA score	9.48	0.002	−0.13	−0.22	−0.049

Fixed effects (*df*1 = 1, *df*2 = 924). AMMSA score = Scores on the 11-item short version of the Acceptance of Modern Myths About Sexual Aggression scale (Gerger et al., 2007). ^a^ The coefficient value indicates the increase of the rating per score-increase of 1 (e.g., unpleasantness rating decrease of −0.13 per AMMSA score increase of 1).

**Table 3:** Influences on the proportion of dwell time spent looking at the body.

Independent Variable	*F*	*p* Value	Coefficient ^a^	95% Confidence Interval	
				Lower Limit	Upper Limit
Condition	12.45	<0.001	−7.85	−11.48	−4.22
Gender	1.34	0.247	−4.84	−7.94	−1.74
Gender × Condition	8.36	0.004	6.92	2.22	11.61
OOS score	23.90	<0.001	0.30	0.18	0.42
AMMSA score	31.06	<0.001	2.96	1.92	4.00

Fixed effects (*df*1 = 1, *df*2 = 834). OOS score = Scores on the Other-Objectification questionnaire (Strelan and Hargreaves, 2005). AMMSA score = Scores on the 11-item short version of the Acceptance of Modern Myths About Sexual Aggression scale (Gerger et al., 2007). ^a^ The coefficient value indicates the increase of dwell time on the body per score-increase of 1 (e.g., dwell time increase of 2.96 per AMMSA score increase of 1).
